# Systematic Review of Cost-Effectiveness of Injury Prevention Interventions in Soccer—Evidence Why Health Agencies Should Address It

**DOI:** 10.3390/ijerph182211901

**Published:** 2021-11-12

**Authors:** Monika Grygorowicz, Martyna Wiernicka, Marzena Wiernicka

**Affiliations:** 1 Department of Physiotherapy, Poznan University of Medical Sciences, 61-701 Poznan, Poland; 2 Rehasport Clinic FIFA Medical Centre of Excellence, Sports Science Research Group, 60-201 Poznan, Poland; 3 Private Physiotherapy Practice, 60-271 Poznan, Poland; martynawiernicka@gmail.com; 4 Department of Kinesiotherapy Developmental Physiotherapy, Faculty of Health Sciences, Poznan University of Physical Education, 61-871 Poznan, Poland; wiernicka@awf.poznan.pl

**Keywords:** soccer, football, cost-effectiveness, injury prevention, health care system

## Abstract

Soccer injuries are a recognized problem worldwide. Several injury prevention programs have been confirmed to reduce the number of injuries in female and male players. Unfortunately, there is a lack of data about their cost, burden, and benefit for the health care system. In this paper we aim to systematically review the literature and critically evaluate the economic quality of injury prevention interventions implemented across different populations of soccer players. Web of Science, Medline, SPORTDiscus, Ovid, and other databases were searched from January 2011 through July 2021. Research articles were only selected for analysis if they focused on the cost-effectiveness of injury prevention, were experimental papers written in English, and were published following the peer-review process. Three cluster RCT and one retrospective study met the criteria. Cost data on incremental cost-effectiveness ratios (ICERs) were extracted. The included studies had a good/average quality of economic evaluation. Based on ICERs, injury prevention interventions were cost-effective in three out of the three comparisons. One study did not report the ICER value. However, since economic analyses were reported with varying methodological approaches and results, more data are required to recognize the cost-effectiveness of soccer-specific injury prevention interventions and their benefit for the health care system.

## 1. Introduction

Sport and physical activity are related to a higher risk of injury occurrence [1]. Various injury prevention strategies have been implemented across different disciplines to reduce the number of sport-related injuries [2,3,4,5]. Apart from its health- and sport-related value [6], the introduction of injury prevention programs significantly reduces the costs of sports injuries [2,4]. In 2005 Verhagen et al. published one of the first studies dedicated to evaluating the cost-effectiveness of proprioceptive training. The training was performed on balance boards, and it aimed to prevent ankle sprains among volleyballers. Sensitivity analysis confirmed that this intervention might be cost-effective over a longer period when applied among athletes with previous ankle injuries [2]. The importance of cost-effective interventions for preventing sports-related injuries was documented previously [7].

Several soccer-specific injury prevention programs have been developed and implemented across different athletes. Considering the beneficial effects of these programs on the personal and social level, injury prevention interventions can be identified as an essential tool for minimizing the risks associated with sport [8].

However, until now, no review on the cost-effectiveness of soccer-specific injury prevention programs has been conducted, even though it has been widely confirmed that such interventions significantly reduce the risk of injuries [9,10], enhance players’ performance [11,12], and by maintaining players ready to play—they can affect sports results [13]. Health/government agencies will only take relevant actions when there is convincing data that the problem to be addressed is significant at the population level [14]. This systematic review may bridge the knowledge gap, provide evidence and relevant information to health agencies, and explain why the authorities should devote more financial support in a public health approach to soccer-specific injury prevention programs. It has to be underlined that economic analysis should be key in setting health care priorities, given that these types of evaluation are the source of information on procedures with the most satisfactory balance between expenses and health benefits [15].

Usually, three main types of analysis are used to present economic evaluations when comparing different injury prevention interventions. Cost-effectiveness analysis, cost-utility analysis, and cost-benefit analysis might be applied when comparing at least two different interventions [16,17]. In cost-effectiveness, the costs of compared interventions are measured by monetary expenditures. Health outcomes are expressed in natural units, such as life-years gained or the number of adverse events avoided, e.g., sports injuries, due to a prevention program [16]. The primary outcome of cost-effectiveness analysis is the incremental cost-effectiveness ratio (ICER). By definition, an ICER is a difference between the costs of providing the competing interventions divided by the difference in effectiveness (i.e., the number of injuries prevented). In cost-utility analyses, health benefits are measured by a quality-adjusted life year, and for cost-benefit analyses, in monetary units [17].

To the best of our knowledge, there is no systematic review of economic evaluations published for injury prevention programs in soccer. It is necessary to summarize findings from the research literature to show which methodological attitudes to cost analysis are presently being used in papers regarding soccer-specific injury prevention strategies. Therefore, this study aims to delineate the outcomes of a systematic review of the relevant studies and critically evaluate the economic quality of injury prevention interventions implemented in various populations of soccer players.

## 2. Materials and Methods

Before the review process, we established the study protocol following the Reporting Items for Systematic Reviews and Meta-Analyses (PRISMA) reporting guidelines [18].

### 2.1. Search Strategy

Between 1 and 7 July 2021, we searched several databases, including Web of Science, Medline (EBSCOhost) (EBSCO Industries, Inc International Headquarters, Birmingham, Alabama, USA), SPORTDiscus with Full Text, Academic Search Ultimate, Health Source: Nursing/Academic Edition, OpenDissertations, Teacher Reference Center, MasterFILE Premier, Business Source Ultimate and Ovid MEDLINE(R) ALL. These databases are essential bibliographic data sources covering both medical and sports aspects, including physical fitness, exercise, sports medicine, sports science, physical education, kinesiology, coaching, training, biomechanics, movement science, injury prevention rehabilitation, or physical therapy. We searched at least four databases following the suggestion by Bramer et al. that a systematic review requires four databases to ensure adequate coverage of the review topic [19].

Table 1 lists the specific keywords and Boolean operators that we applied to search each database using the indicated research platform access. No language limit was applied in the search strategy. We searched databases from January 2011 through July 2021.

### 2.2. Selection Criteria

The study aimed to analyze the cost-effectiveness of injury prevention programs implemented across the soccer community, and papers were qualified for inclusion when they met the following criteria: (1) participants: soccer players included in all levels of sport participation; (2) intervention and comparator: studies comparing at least two different injury prevention programs; (3) outcomes: studies reporting economic evaluations including cost analyses comparing no less than two injury prevention programs, based on cost-effectiveness and/or cost-utility and/or cost-benefit analysis; (4) study type: only experimental papers, written in English, and published following the peer-review process. Thus, according to the PICO strategy, we used “football OR soccer” for participants, “injury AND prevention” for intervention and comparator, and “return of investment” OR ROI OR “cost-benefit” OR cost * OR “cost effective *” OR cost-effectiv* for outcomes.

### 2.3. Data Extraction

The process of extracting the relevant papers included a three-phase screening process: investigating the titles (phase 1), abstracts (phase 2), and full-text paper (phase 3). Two independent raters (first and second authors: M.G. and M.W.) performed the screening process. The raters discussed any potential difference on papers’ suitability to find an agreement. Methodological details, including participants, implemented injury prevention program and comparator, time frame, and economic outcomes, were extracted into the summary Table 2.

### 2.4. Quality Assessment

We used the Drummond checklist to assess the papers in terms of their economic quality [15]. The Drummond checklist was developed as an instruction tool for the critique of economic assessment, and it includes the following main points: 1—defining of the research question; 2—the comprehensive explanation of the study and/or alternatives applied in different research groups; 3—the effectiveness of the program; 4—the recognition, 5—measurement, and 6—credible valuation of costs and consequences; 7—calculation of any costs and consequences adjusted for differential timing; 8—incremental analysis; 9—estimating including the allowance for uncertainty and sensitivity analyses; and 10—presentation and discussion of study results taking into account all issues of concern to users and present scientific background. We used a rating scale described by Doran [22] to assign possible points to each criterion. The checklist was filed against all evaluated criteria by two reviewers (first and last authors: M.G and M.W.). The total scores present an economic quality appraisal of poor (1–3 points), average (4–7 points), and good (8–10 points) [23].

## 3. Results

### 3.1. Literature Search

**Figure 1:**
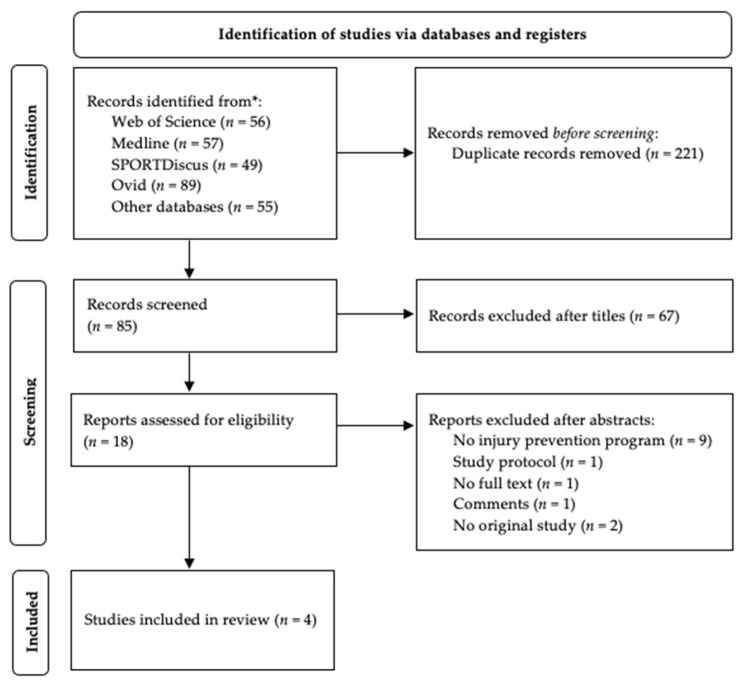
Flow diagram of the number of selected and included studies.

The database search revealed 56 results in Web of Science, 57 titles in Medline (EBSCOhost), 49 papers in SPORTDiscus with Full Text, 36 research in Academic Search Ultimate, five titles in Health Source, and five results in OpenDissertations, four papers in Teacher Reference Center, three titles in MasterFILE Premier, two research in Business Source Ultimate and 89 titles in Ovid MEDLINE(R) ALL (Figure 1). After deduplication of titles, 85 papers were selected for further examination. Eighteen papers seemed to match the selection criteria and were selected after screening the titles. However, after further analysis of abstracts, 14 papers did not meet the inclusion criteria and were excluded for the following reasons: the paper did not evaluate an injury prevention program (*n* = 9), the study described the study protocol for an on-going trial (*n* = 1), study available only as an abstract (*n* = 1), the study was a comment to original work (*n* = 1), and the study was designed as a systematic review (*n* = 2). Finally, four papers met the inclusion criteria and were selected for the present systematic review.

### 3.2. Study Description

Three included studies were conducted in three different countries: the Netherlands [3], Canada [21], and Spain [23]. One study [4] was designed as a multinational project, and it involved four countries (Switzerland, The Netherlands, Germany, and the Czech Republic). Only one study [4] involved a middle-income country, whereas five remaining countries in this study were classified as high-income countries.

Three different injury prevention strategies were assessed. The “FIFA 11” injury prevention program [3,23] (named as the “FIFA 11” by Nauni-Garcia et al. [23] and “The11” by Krist et al. [3]) included 10 exercises and the fair play rule. The exercise focused on core stabilization (“the bench,” “sideway bench” and “cross-country skiing”), eccentric training of hamstring (called “Russian” or “Nordic” hamstrings), proprioceptive training (“chest-passing in single-leg stance,” “forward-bend in single-leg stance” and “figure-of-eight in single-leg stance”) and dynamic stabilization with plyometric and straight leg alignment (“jumps over a line,” “zigzag shuffle” and “bounding”). The neuromuscular training (NMT) program described by Marshall et al. [16] included neuromuscular training components (e.g., eccentric exercises of hamstring muscle strength, walking lunges eccentric quadriceps exercise, core stability abdominal strength exercise, single-leg jumps concentrating on proper position and core stability and team-based balance exercise) and aerobic and dynamic stretching exercises, additionally to the home-based balance training program (performed on a 16-inch diameter wobble board). The “11+ Kids” evaluated by Rössler et al. 2018 [4] consisted of seven different exercises. Three of them aimed at unilateral, dynamic stability of the legs (”jog and look at the coach (to stop),” “skating hop” and “one leg stance”), three were dedicated to the whole body, and trunk strength and/or stability (“push up,” “one leg hops” and “spiderman”), and one exercise focused on the proper technique of fall (“rollover”). The economic aspects of the implementation of these interventions were assessed across male amateur players aged 18–40 years [3,23], male and female youth players aged 13–18 years [21], and boys and girls aged 7–12 years [4].

None of the studies used any discount rate to calculate the cost-effectiveness of the injury prevention program. No studies reported information on life years gained.

### 3.3. Assessing the Quality of Economic Evaluation

All the studies that focused on analyzing the cost-effectiveness of the implemented injury prevention strategies were rated average or good (Table 3). Additionally, all studies presented data on cost–benefit analysis. The research question was clearly stated in each study. Cost-effectiveness analysis was performed in each study, comparing direct healthcare costs of the “FIFA 11” and NMT and “11+ Kids” group with costs in a non-intervention group, appropriately chosen, who performed their standard warm-up. The intervention strategies and alternative interventions were described in sufficient detail in each paper.

Cost components and costing sources were sufficiently reported in each study, including the costs of intervention and direct and indirect costs of healthcare.

Two studies [3,4] conducted an economic analysis from a social perspective, considering all costs related to the player’s injury, regardless of who would cover them. However, in the study by Krist et al., direct non-healthcare expenses (e.g., travel costs or the costs of the patient’s time or the time of family members) were not included in the economic evaluation [3]. Two studies also applied cost analysis from a healthcare perspective, using standardized medical fees in line with the national medical organization data [4] or costs were divided into public (i.e., direct healthcare system’s costs) and private costs (together with all treatment-related out-of-pocket expenses covered by players and their families) [21]. One study reported it was impossible to complete the evaluation from a societal perspective due to the lack of relevant data [21]. One study did not provide thorough information on the study perspective [23].

The period of study covered one soccer season in three studies [3,4,21]. Only one study precisely stated that this period ranged from August to June [3]. One study analyzed the indoor soccer season [21], and in one study, the study horizon was stretched across two soccer seasons [23].

Three papers were designed as cluster-randomized trials [3,4,21], and one study was an analytical observational retrospective cohort study [23].

### 3.4. Costs

Costs can be differentiated into several categories: direct costs of medical procedures (e.g., hospital stay), indirect medical costs (e.g., costs of care during life years gained), direct non-medical costs (e.g., costs of travel), and indirect non-medical costs (e.g., productivity loss) [24]. Direct costs in the analyzed studies covered: visits to physicians [3,4,21,23], emergency departments [3], surgeries [3], hospital stays [3], X-rays [3,4,21], scans and casts [3,21] or diagnostic tests [23], healthcare costs, usually paid by relatives of injured players, classified as out of pocket healthcare costs (e.g., physiotherapy [3,21,23], and visit to a chiropractor [4,21]), manual therapist [3], medical specialists [3], athletic and massage therapy [21], acupuncture [21], medication [3], splints [21], braces [3,4,21], crutches [3,21], tensors [21] and secondary preventive devices (e.g., tape, insoles, groin pants) [3].

Direct intervention costs comprised payments for published guidebooks [4] or information packages [3] and the arrangement of instruction courses dedicated for soccer coaches [4], or a monthly salary of physiotherapists included in the intervention [23], or costs of demonstration and evaluation session [3] and the possible costs of coaches participating in the “11+ Kids” instructional course [4]. Moreover, when additional equipment was used during the injury prevention program, the direct intervention costs also included buying such equipment, e.g., exercise mats [3] or wobble boards [21].

Direct costs related to the nationwide implementation of the “11+ Kids” injury prevention in Switzerland consisted of expenses for published guidebooks and releasing the “11+ Kids” manual [4], costs for “11+ Kids” educational courses [4], and costs of developing, launching and maintenance of program’s website [4].

Indirect costs related to the loss of productivity of players and parents or guardians due to reduced working capacity (e.g., parents or guardians’ productivity loss due to taking care of the injured child) were not considered in two studies [4,21]. One study included absenteeism from paid work and absenteeism from school, which caused loss of productivity, as indirect costs [3]. Lost wages and school absenteeism were included as indirect injury-related costs analysis in one study [23].

### 3.5. Injury Prevention Programs Costs

Three of the four studies presented injury prevention costs. Krist et al. analyzed the costs of the FIFA 11 injury prevention program. The accumulated costs of the intervention program were calculated at €287 per team, which correspond to €14.14 per player. Since 236 participants were included in the group where a soccer-specific injury prevention program was applied, the total costs of the injury prevention program were equal to €3337 [3]. Marshall et al. evaluated the neuromuscular training intervention. In this study, injury prevention program costs consisted of wobble board costs ($20 per participant) and costs of the training session ($35 per hour × 2 h per team). Thus, overall intervention costs were $9831) [21]. The third program, dedicated to children, was investigated by Rössler et al. [4]. Intervention costs in this study were determined in two ways: as costs calculated in the research and as costs estimated for a countrywide realization of the “11+ Kids” injury prevention program. Total costs in the study were CHF 2467, and in the countrywide implementation scenario, CHF 568,533. The comprehensive intervention cost calculated in the study was CHF 4.02 per player, and if implemented countrywide, it was CHF 1.94 per player [4].

### 3.6. Injury Prevention Interventions and Their Incremental Cost-Effectiveness Ratio

The incremental cost-effectiveness ratio (ICER) was estimated to summarize the cost-effectiveness of injury prevention strategies in three studies. However, different units were used for the ICER calculation. Rössler et al. defined the ICER as the difference in cost per player between the intervention and control groups divided by the difference in the number of injuries per player between the intervention and control groups [4]. Marshall et al. calculated the ICER as the difference in costs per 1000 player hours and per 100 players between intervention and standard warm-up practice (control group) and the difference in the effect between intervention and control [21]. Krist et al. calculated ICER by dividing the difference in mean total costs per participant between the intervention group and control group by the difference in numbers of injuries between the two groups, corrected for the difference in the number of participants between the groups [3]. The study by Nouni-Garcia et al. [23], which also analyzed costs reduction due to FIFA 11 program implementation, did not calculate the incremental cost-effectiveness ratio.

### 3.7. Cost-Effectiveness Analysis

All four studies considered soccer-specific injury prevention programs as cost-effective as the standard warm-up implemented in male and female soccer players. Krist et al. analyzed the cost-effectiveness of the FIFA 11 injury prevention program. They calculated that mean overall costs per player were less among the players who participated in the injury prevention group when compared to players from the control group (€161 vs. €361, respectively). Significantly lower costs (over €350, 95% CI €51–733) were reported per injured player in the intervention group. Based on bootstrap analyses, the FIFA 11 program was confirmed to be more effective and cost-saving in 55% and less effective and cost-saving in 43%. When sensitivity analysis was applied, the intervention program tuned out to be cost-saving and more effective in 55% of the bootstrap replicates and cost-saving and less effective in 45% [3]. In the study by Nouni-Garcia et al. [23], the authors’ calculated savings per player for two seasons and the return of each euro invested in the injury prevention program. The mean saving per player in the FIFA 11 group was €924 for two seasons or €462 per player per season. Hence, the overall savings were €9766.68, and the total projected costs that could be avoided were calculated at €38,892, meaning that €3.98 was obtained in return for each euro invested [3].

For neuromuscular training, cost-effectiveness analysis was performed by Marshall et al. [21]. Over 90% of 10,000 bootstrap iterations showed that the neuromuscular training intervention (NMT) was less costly and more effective when compared to a typical warm-up. Authors estimated that incremental costs, expressed as the difference in costs between an NMT intervention and standard exercises (warm-up), were at $−1099/1000 player hours (95% CI $−2145 to $−180). Similar patterns were confirmed when the difference in costs/100 players was estimated [16]. When analyzing healthcare costs, cost reduction of more than 43% was observed in youth soccer male and female players who participated in neuromuscular training intervention compared to those who performed only standard warm-up. The authors also estimated that 4965 injuries and over $2.7 million in healthcare costs could have been avoided just in one season if neuromuscular interventions had been implemented across 58,100 youth soccer players in the Alberta region. Additionally, the sensitivity analysis revealed that over $4.2 million less could have been spent by the healthcare system if comparable NMT intervention (without considering the intervention costs of wobble boards or training sessions) had been applied as the prevention program in just one soccer season [21].

Rössler et al. performed the cost-effectiveness analysis for the “11+ Kids” injury prevention program. When compared to the control group, the ICER for the intervention group was dominant when analyzing the difference of CHF −23.12 in the mean cost per player and 7.9% in the mean efficacy of the injury prevention intervention. When compared with players who did not participate in a soccer-specific injury prevention intervention, 59% lower costs of healthcare per player season and 51% lower costs per 1000 h of soccer exposure (CHF −23.12, 95% CI −39.09 to −7.14 and CHF −240.66, 95%CI −406.89 to −74.32, respectively) was observed in the “11+ Kids” intervention group. Of all bootstrapped ICERs, 94.6% indicated that substantially lower costs and fewer injuries were recorded among children performing the intervention program than participants from the control group [3]. For a countrywide implementation scenario, considering the 7.9% difference in efficacy and the CHF −23.12 difference in the mean cost per player, 95.5% of the bootstrapped ICERs demonstrated the dominance of the “11+ Kids” group over the standard warm-up group [4]. The author also estimated costs that could have been avoided by implementing the “11+ Kids” in Switzerland. Based on the total number of players in Switzerland and the difference in the mean cost per player (CHF −25.50) between the intervention group and the control group, it was calculated that in only just one season, CHF 1.48 million less could have been spent by the healthcare system if the “11+ Kids” injury prevention strategy had been implemented countrywide [4].

## 4. Discussion

Our study aimed to identify and critically evaluate the quality of economic analysis of soccer-specific injury prevention programs implemented across different populations of players. The four articles that met the criteria for inclusion showed favorable findings of the cost-effectiveness of soccer-specific injury prevention programs as compared to standard warm-up.

The studies presented three different injury prevention programs: “11+ Kids” implemented across girls and boys (aged 7–12) [4], a program for the youth female and male population (aged 13–18 years) [21], and the intervention for male amateur soccer players (aged 18–40 years) [3,23]. These studies provide data on cost-effectiveness for soccer populations at the grassroots level. It is estimated that over 300 million people play soccer worldwide [25], and only 1% of them represent the highest professional level of sport participation. Participation in soccer at the amateur or recreational level is very common, and thus the probability of injuries is high. However, evidence shows that reduction of injury incidence translates into lower socio-economic and health-related costs, and due to the sheer size of the soccer community, prevention programs could benefit the entire health care system.

As far as we know, it is the first systematic review that critically investigated the quality of studies evaluating the economic aspects of soccer-specific injury prevention area. Previous reviews assessed the economic studies dedicated to the cost-effectiveness of injury prevention programs covered more wide-ranging issues. For example, Polinder et al. prepared the systematic review focused on evaluating the economic papers on injury prevention, including home and leisure injuries, traffic-related injuries, occupational injuries [24]. The only two interventions applied in the athlete population were dedicated to analyzing the cost-effectiveness of the tailored intervention to prevent ankle sprains [2] and using face protection for hockey players [26]. None of the analyzed studies focused on soccer. A few thorough economic analyses associated with sport and recreation injury prevention interventions have been reported in the systematic review published by Michaels-Igbokwe et al. [21]. It was confirmed that, in one season, savings of $499 per 1000 player hours could be achieved due to fewer injuries, and 90% of simulated estimates revealed that neuromuscular training could be less costly and at the same time more effective when compared to standard exercise (warm-up) [21].

The type, localization, and severity of injuries strongly differ. Thus, previous studies concluded that it might be very challenging for decision-makers to include injury strategies in a policy framework since they chose between different types of injury prevention or health promotion strategies based on uncommon measures of intervention outcomes [27].

Many studies have demonstrated the effectiveness of various injury prevention programs in both male and female soccer players at various levels of the sport. Studies included in this review also evidence the cost-effectiveness of these programs related to the direct health care costs associated with these injuries. Unfortunately, we cannot provide broader summaries based on the papers reviewed. The main reason for this is that the ICER and cost-effectiveness plans used in the three papers were not based on the same units. Marshal et al. used a cost/100 and cost/1000 player training hours [21]. Krist et al. used ICER, but training/match hours were not considered [3]. Similarly, Rössler et al. expressed ICER as a difference in the number of injuries per player between the intervention and control groups [4]. Still, they did not include exposure hours in this calculation. In the future, it would be helpful to use the same measures to determine the cost-effectiveness of given programs to make a summary case for the widespread introduction of such programs into the health care system.

Our review focused on soccer-specific injury prevention strategies that have been extensively analyzed for over 20 years. We believe that the information on cost-effectiveness presented in this review in an aggregated way, with incremental analysis, might be relevant enough to illustrate that soccer-specific injury prevention is a meaningful public health matter and should be tackled more systemically. Cost-effectiveness analysis gathered in this systematic review provides meaningful arguments why health agencies should address it, developing countrywide systemic in-jury prevention strategies for the most played and most popular game in the world. Where injury prevention programs have been demonstrated to reduce the number and severity of injuries and save costs, wider implementation and upscaling should be a priority [27].

However, a few concerns that limit the usefulness of the findings must be considered when analyzing the aggregated data on the cost-effectiveness of injury prevention programs in this review. Wide methodological approaches were reported, including different perspectives, project time frames, study designs, and different costs or outcomes categories. Two papers did not implement the societal perspective, not identifying relevant costs and consequences [4,23], and one study did not report incremental cost-effectiveness analysis [23]. Additionally, although we tried to include several databases in the literature search process, it might be possible to find more relevant studies through different databases or in unpublished grey literature, the existence of which we are not aware. Moreover, since we included only peer-reviewed academic journals, some studies published in books may have been overlooked. We used the Drummond checklist in our methodology approach. Although it is a recognized tool for evaluating the economic quality of papers, it is not a standardized assessment scale. It could be identified as a limitation of this study.

## 5. Conclusions

This study concisely presented the results and quality characteristics of four studies dedicated to the economic evaluation of soccer-specific injury prevention programs. The essential value of our paper lies in systematically gathering the data from different research. As a result, scientists, practitioners, health agencies, and policymakers could refer to this information in an aggregate form. Although some methodological limitations must be considered, overall quality of economic evaluation of these studies was good and average. The existing economic evaluations of “FIFA 11”, neuromuscular training, and “11+ Kids” injury prevention programs are encouraging for injury prevention specialists. They could raise the likelihood that economic evidence is incorporated into the health policymaking process associated with the systemic implementation of injury prevention among children, female, and male soccer players in different countries. However, it must be highlighted that some relevant limitations arise from the limited number of studies included in this systematic review and their methodological differences.

## Figures and Tables

**Table 1:** The keywords and Boolean operators used in our search strategy.

Database	Research Platform	Search Strategy
Web of Science *	Web of Science Platform	**TS** = **(**“return of investment” OR ROI OR “cost benefit” OR cost * OR “cost effectiv *” OR cost-effectiv *) AND TS = (football OR soccer) AND TS = (injury AND prevention)
Medline (EBSCOhost) **	EBSCOhost Research Platform	**AB** = **(**“return of investment” OR ROI OR “cost benefit” OR cost * OR “cost effectiv *” OR cost-effectiv *) AND AB = (football OR soccer) AND AB = (injury AND prevention)
SPORTDiscus with full text **		
Academic Search Ultimate **		
Health Source: Nursing/Academic Edition **		
OpenDissertations **		
Teacher Reference Center **		
MasterFILE Premier **		
Business Source Ultimate **		
Ovid MEDLINE(R) ALL ***	Ovid Research Platform	(“return of investment” OR ROI OR “cost benefit” or cost * OR “cost effectiv *” OR cost-effectiv *).ab,kw,ot,sy,ti AND (football OR soccer).ab,kw,ot,sy,ti AND (injury AND prevention).ab,kw,ot,sy,ti

* TS—topic field in advanced search query builder was searched using the specific keywords. ** AB—abstract field in advanced search query builder was searched using the specific keywords. *** ab, kw, ot, sy, ti—abstract, keyword heading, original title, synonyms and title fields in advanced search query builder were searched using the specific keywords.

**Table 2:** Studies’ characteristics.

Scheme	Study Design	Participant	Intervention	Comparator	Time Frame	Injury Definition	Outcomes	Perspective, Type of Currency, Time Period Costs were Measured	Sensitivity Analysis	Discounting, Time Horizon
Nouni-Garcia et al. 2019 [20]	Retrospective cohort study	Male amateur players aged 18–40 years INT: *n* = 42 CON: *n* = 42	The “FIFA 11” intervention, 2× week	Usual training	Two soccer seasons	All time-loss lateral ankle ligament and hamstring injuries that had occurred during training sessions and competitions	mean total cost per player for the two seasons	Not provided, EUR, 2008–2021	No	Not applicable
Krist et al. 2013 [3]	Cluster RCT	Male amateur players aged 18–40 years INT: *n* = 223 CON: *n* = 233	“The11” injury prevention program during the warm-up (10 exercises and advice regarding fair play) 2 or 3× week for 33 weeks	Regular warm-up exercises, which usually consists of running exercises, dynamic and static stretching, and sprinting.	Soccer season	Physical complaint sustained by a participant that resulted from a soccer training session or soccer match, irrespective of the need for medical attention or time lost from soccer activities	intervention costs costs associated with the implementation of the preventive exercises costs of productivity losses due to absence from work ICER	Societal, EUR, 2009	One-way using a range of estimates of cost items for injury prevention program	Not applicable 1 year
Marshall et al. 2016 [21]	Cluster RCT	male and female, ages 13–18 years INT: *n* = 380 CON: *n* = 364	Fifteen-minute neuromuscular training in intervention group including 10 min of neuromuscular training components (e.g., strength, agility, balance) and 5 min of aerobic and dynamic stretching components, in addition to a 15 min home-based balance training (on a wobble board).	Standard of practice 15 min warm-up routine including aerobic, static stretching and dynamic stretching components and a home program, including only stretching components	Indoor soccer season + 6 months following the end of the season INT: h = 24,051 h of athlete participation CON: h = 23,597 h of athlete participation	Soccer-related injuries that required medical attention resulted in the inability to complete a session or in time loss from play	cost of injuries/1000 player hours cost of injuries/100 players mean cost per injury intervention costs ICER	Not stated, $, 2006–2007	A sensitivity analysis was conducted in which the intervention costs (wobble boards and training session) were excluded from the total cost for the training group	Not applicable 1 year
Rössler et al. 2018 [4]	Cluster RCT	boys and girls, aged 7–12 years INT: *n* = 614, aged 11 (1.2) CON: *n* = 388 aged 10.6(1.1)	The “11+ Kids”, fifteen-minute injury prevention program at the beginning of each training session throughout the season	Regular warm-up program	Soccer season from August to June INT: h = 43,777 h of athlete participation CON: h = 32,596 h of athlete participation	Any soccer-related injuries that resulted in at least one of the following: (a) inability to complete the current match or training session and/or (b) absence from subsequent training sessions or matches and/or (c) injury requiring medical attention.	direct healthcare costs intervention costs intervention costs per player costs of a nationwide implementation of “11+ Kids” in Switzerland mean cost per player cost of injuries/1000 h of soccer ICER	Societal, EUR, 2014–2015	Sensitivity analysis was performer by cutting the respective time period (exposure time and injury events) in the CON group at the beginning of the season	Not applicable 1 year

RCT—randomized controlled trail, INT—intervention group, CON—control group, ICER—incremental cost-effectiveness ratios.

**Table 3:** Evaluation of economic criteria for evaluating study quality using the Drummond checklist.

Criteria	Rössler 2018	Marshall 2016	Krist 2013	Nouni-Garcia 2019
Research question well defined?	yes	yes	yes	yes
2. Comprehensive description of alternatives?	yes	yes	yes	yes
3. Effectiveness of program established?	yes	yes	yes	yes
4. Important and relevant costs and consequences for each alternative identified?	yes	yes	yes	yes
5. Costs and consequences measured accurately and appropriately?	yes	yes	yes	yes
6. Costs and consequences valued credibly?	yes	yes	yes	yes
7. Costs and consequences adjusted for differential timing?	yes	yes	yes	no
8. Incremental analysis of costs and consequences performed?	yes	yes	yes	no
9. Allowance made for uncertainty in estimates (sensitivity analysis)?	yes	yes	yes	no
10. Presentation and discussion of study results include all issues of concern to users?	yes	yes	yes	yes
Total score	Good	Good	Good	Average

yes—when the criteria were fulfilled; no—when the criteria were not fulfilled.

## Data Availability

Data are available from the corresponding author upon due request.
